# *Agrobacterium*-Mediated Transformation of Tomato with rolB Gene Results in Enhancement of Fruit Quality and Foliar Resistance against Fungal Pathogens

**DOI:** 10.1371/journal.pone.0096979

**Published:** 2014-05-09

**Authors:** Waheed Arshad, Ihsan-ul- Haq, Mohammad Tahir Waheed, Kirankumar S. Mysore, Bushra Mirza

**Affiliations:** 1 Department of Biochemistry, Faculty of Biological Sciences, Quaid-i-Azam University, Islamabad, Pakistan; 2 Department of Pharmacy, Faculty of Biological Sciences, Quaid-i-Azam University, Islamabad, Pakistan; 3 Plant Biology Division, The Samuel Roberts Noble Foundation, Ardmore, Oklahoma, United States of America; National Taiwan University, Taiwan

## Abstract

Tomato (*Solanum lycopersicum* L.) is the second most important cultivated crop next to potato, worldwide. Tomato serves as an important source of antioxidants in human diet. *Alternaria solani* and *Fusarium oxysporum* cause early blight and vascular wilt of tomato, respectively, resulting in severe crop losses. The foremost objective of the present study was to generate transgenic tomato plants with *rolB* gene and evaluate its effect on plant morphology, nutritional contents, yield and resistance against fungal infection. Tomato cv. Rio Grande was transformed via *Agrobacterium tumefaciens* harbouring *rolB* gene of *Agrobacterium rhizogenes*. *rolB*. Biochemical analyses showed considerable improvement in nutritional quality of transgenic tomato fruits as indicated by 62% increase in lycopene content, 225% in ascorbic acid content, 58% in total phenolics and 26% in free radical scavenging activity. Furthermore, *rolB* gene significantly improved the defence response of leaves of transgenic plants against two pathogenic fungal strains *A. solani* and *F. oxysporum*. Contrarily, transformed plants exhibited altered morphology and reduced fruit yield. In conclusion, *rolB* gene from *A. rhizogenes* can be used to generate transgenic tomato with increased nutritional contents of fruits as well as improved foliar tolerance against fungal pathogens.

## Introduction

Tomato is an important cultivated crop that is economically attractive for medium-scale farmers due to its high yield and relatively short duration. Across the globe, in 2008, tomato production was about 136.230 million tonnes from an area of 4.837 million hectares with an average yield of 28.16 tonnes/ha [1]. Tomato fruit contains variety of compounds including antioxidants such as lycopene and ascorbic acid, which are important for human health.

Tomato crop production is affected by a number of biotic factors including viruses, bacteria, fungi and nematodes causing devastating diseases resulting in great economic losses [2]. Early blight and *Fusarium* wilt are two very important and devastating diseases of tomato. Early blight, also known as *Alternaria* leaf spots, is a common disease caused by the fungus *A. solani*, while *Fusarium* wilt is caused by the soil dwelling fungus *F. oxysporum* f. sp. *lycopersici*. Various methodologies have been developed to lower these crop losses by different diseases to ensure the food supply. Development of disease resistant plants could be the most efficient practice with less hazards [3].

In addition to modulatory role in cell differentiation and plant growth, *rol* genes from *Agrobacterium rhizogenes* are the most probable activators of secondary metabolism in transformed cells in diverse plant families including Vitaceae, Solanaceae, Rosaceae, Araliaceae and Rubiaceae [4]. The exact mode of action of *rolB* gene is not clearly understood. Various researchers have reported different findings regarding *rolB* expression. It has been reported that *rolB* transformed calli of *Rubia cordifolia* showed increase in the activity of isochorismate synthase (*ICS*) gene which in turn enhanced the production of anthraquinones [5]. RolB protein, β-glucosidase, enhances the concentration of active and free indole acetic acid (IAA) by its release from inactive conjugates of glucose [6]. Conversely, IAA-glucosides are not the *rolB* substrates in plant tissues [7]. However, they increase auxin sensitivity in the *rolB* transgenic cells [8], [9]. The auxin sensitivity also depends on increased tyrosine phosphatase activity in *rolB* transformed cells that distracts the signal transduction pathway of the hormone [10]. Such transduction and sensitivity alter the physiological pattern of the transformed cells and ultimately the whole plant [11].

The *rolB* gene plays a primary role in plant morphogenesis by functioning as a meristem stimulating gene for a variety of organs including shoots, roots and flowers [12]. Generally, the *rolB* gene in transformed plants is associated with the increased or decreased apical dominance, wider leaves, small to large flowers, reduced flower development and flower induction, low pollen viability, enhanced rooting and stunted phenotype. It has been established that the *rolB* gene influences the level of endogenous hormones but these dissimilar observations point towards the unpredictable effect of *rolB* gene in the transformed plants [13]. Expression of *rolB* gene has been reported in a number of plant species including tobacco [14]–[17], kalanchoe, carrot [14], [18], [19], *Antirrhinum* [20], woody plants [13], soybean [21] and tomato [22], [23]. Most of these studies concerning the expression of *rol* genes are related to growth and development of plants. However, the effect of these genes in relation to the improvement of nutritional contents of tomato fruit has not been investigated to date.

The aim of the present study was to investigate the effects of *rolB* gene expression in tomato plants in terms of fruit quality. Changes in plant morphology were observed in tomato plants expressing *rolB* gene. The fruits of transgenic tomato plants exhibited higher nutritional quality and the tomato plants showed foliar tolerance to two fungal pathogens *A. solani* and *F. oxysporum*.

## Materials and Methods

### Plant material

The seeds of tomato variety Rio Grande, purchased from the local market, were surface sterilized with 5% (w/v) sodium hypochlorite (NaOCl) solution adding one drop of Tween-20 for 10–15 min with continuous shaking followed by rinsing thrice with sterilized distilled water under aseptic condition. The sterilized seeds were germinated on half strength MS [24] medium (pH 5.8) with 0.8% agar. The Petri plates were kept in dark for four days and later shifted to growth room for 10 days at 25°C under 16 h light and 8 h dark cycle.

### *Agrobacterium* strain and transformation vector

**Figure 1:**
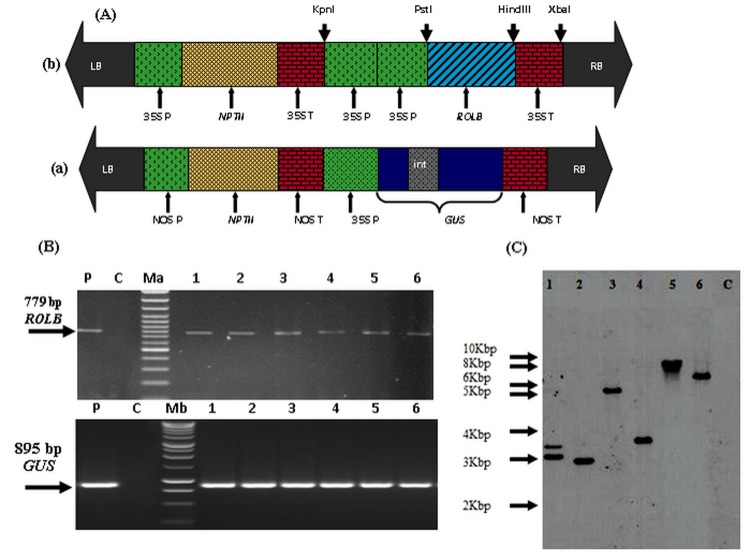
Molecular analysis of *rolB* transformed plants. (**A**) T-DNA region of (a) pLBR30 with *rolB* gene (b) p35SGUSint with *GUS* gene. LB: Left border; 35S P: CaMV35S promoter; 35S T: CaMV35S terminator; NOS P: Nopaline synthase promoter; NOS T: Nopaline synthase terminator; *NPTII*: Neomycin phosphotransferase II gene; *GUS*: β-glucuronidase gene; int: intron; RB: Right border (**B**) PCR amplification of *rolB* gene in *rolB* and *GUS* gene in *GUS* transformed tomato plants cv. Rio Grande. Lanes 1 to 6 =  *rolB* transformed lines RB I to RB VI; Ma  =  DNA Marker (100 bp); Mb  =  DNA Marker (1 kb); P =  Plasmid DNA (+ve control); C =  Untransformed WT control (-ve control) (**C**) Southern blot hybridization of *rolB* transformed tomato plants. Lanes 1–6 =  *rolB* transformed lines RB I to RB VI; C =  Untransformed WT control

*Agrobacterium tumefaciens* strain LBA4404 harbouring binary vector pLBR30 was used for transformation. Plasmids pLBR30 (Figure 1A) contains the *rolB* gene sequences from TL-DNA of *A. rhizogenes*, under the control of double 35SCaMV promoter and 35S CaMV terminator, ligated into the KpnI/XbaI sites of the vector pRT99 [25]. The vector also contains a selectable marker neomycin phosphotransferase II gene (*NPTII*) that confers resistance to antibiotic kanamycin. This vector was provided by Dr. David Tepfer from the Institut National de la Recherché Agronomique (INRA), Versailles, France. In addition, *A. tumefaciens* strain LBA4404 carrying binary vector p35SGUSint (Figure 1A) containing *GUS* reporter gene under CaMV35S promoter was used to generate *GUS* transformed tomato plants which were used as control. This binary vector was kindly provided by Dr. Sarah Grant, Department of Biology, University of North Carolina, Chapel Hill, USA.

### Transformation procedure

A single colony of *A. tumefaciens* from LB agar plates kept at 4°C was picked and inoculated in 50 mL LB broth supplemented with 50 mg/L kanamycin and 10 mg/L rifampicin. The culture was placed overnight at 28°C with continuous shaking at 225 rpm in a shaker incubator. *Agrobacterium* culture was centrifuged and the pellet was resuspended in MS liquid medium (inoculation medium) containing 200 µM acetosyringone and density was set to OD_600_ = 0.75. The cotyledons explants from 10 days old aseptically grown seedlings of Rio Grande were wounded carefully with a fine needle at multiple sites and transferred to the Petri plates having inoculation medium. Total 266 cotyledons were infected for 30 min with *A. tumefaciens* strain LBA4404 harbouring *rolB* gene. Later the explants were co-cultivated for 48 h on co-cultivation medium (CCM: MS medium supplemented with 2 mg/L zeatin +0.1 mg/L IAA) in dark and then transferred to selection medium (SM: MS medium supplemented with 2 mg/L zeatin, 0.1 mg/L IAA, 250 mg/L cefotaxime, 250 mg/L ticarcillin, 100 mg/L kanamycin) after washing with half strength MS liquid medium supplemented with 500 mg/L cefotaxime. The plates were kept in dark for one week at 25±2°C and finally transferred to growth room. The explants with emerging shoot primordia were carefully shifted to shoot elongation medium (SEM: MS medium supplemented with 0.1 mg/L zeatin, 0.1 mg/L IAA, 250 mg/L cefotaxime, 250 mg/L ticarcillin, 100 mg/L kanamycin). The elongated shoots with 3–4 cm length were cultured on rooting medium (RM: MS medium supplemented with 0.05 mg/L IBA and 100 mg/L kanamycin) for root development. The plantlets with well developed roots were then shifted to pots and raised to maturity. The control plants transformed with *A. tumefaciens* strain LBA4404 carrying p35SGUSint were also raised in the same way as described above. The morphological characteristics were observed and recorded for both transformed (*rolB* and *GUS*) and untransformed wild-type (WT) plants. Data for plant height, number of fruits per plant and fruit weight was taken after 16 weeks of sowing whereas the root development was assessed after four weeks.

### Molecular analysis of putative transformed plants

The putative transformants that survived on kanamycin containing medium were subjected to molecular analysis through PCR and Southern blot hybridization in order to confirm the stable transformation. The genomic DNA was isolated from leaves of putatively transformed tomato plants using protocol described by [26]. About 50–100 ng genomic and 10 ng plasmid DNA was used for PCR amplification in 50 µL reaction volume comprising of 1x PCR buffer, 0.2 mM of each dNTP, 2 mM MgCl_2_, 0.25 µM of both oligonucleotide primers and 1.5 U Taq polymerase. PCR reactions were carried out to amplify 779 bp fragment of *rolB* gene by using *rolB* gene specific primers (*RB* F 5′ATGGATCCCAAATTGCTATTCCTTCCACGA-3′ and *RB* R 5′-TAGGCTTCTTTCTTCAGGTTTACTGCAGC-3′) and 895 bp fragment of *GUS* gene by using *GUS* gene specific primers (*GUS* F 5′-AACGGCAAGAAAAAGCAGTC-3′ and *GUS* R 5′-GAGCGTCGCAGAACATTACA-3′). The PCR reactions were subjected to 1 cycle at 94°C for 5 min; 35 cycles at 94°C for 35 sec, 55°C (*rolB*) or 56°C (*GUS*) for 35 sec, 72°C for 45 sec and 1 cycle at 72°C for 10 min.

To confirm integration of the transgene, Southern blot hybridization of transformed plants was performed [27] using the DIG High Prime DNA Labelling and Detection Kit (Roche, Germany). The extracted genomic DNA (20 µg) was digested enzymatically with KpnI (Promega) at 37°C over night and then separated on 0.8% agarose gel. DNA from agarose gel was shifted to a positively charged Hybond N^+^ nylon membrane in the presence of 20x SSC buffer solution (NaCl + Na-Acetate, pH 7). The membrane was hybridized with the probe (Digoxygenin-11-dUTP labelled 779 bp PCR product of *rolB* gene) at 40°C and then washing, blocking and immunological detection on X-ray film using chemiluminescence substrate CSPD were performed.

### Estimation of lycopene content from transgenic tomato fruits

Fully mature red tomatoes were harvested from selected homozygous *rolB* lines along with control plants (*GUS* transformed and WT) and lycopene content was extracted and quantified using protocol described by [28] with some modifications. The tomatoes were ground into a fine paste. About 3.0 g sample was taken in a beaker and 100 mL extraction solution comprising mixture of n-hexane:acetone:ethanol (2∶1∶1; v/v/v) was added to the tomato paste. The homogenate was sonicated for 10 min and 15 mL distilled water was added. In a separating funnel hexane phase was separated and evaporated. The dried sample was resuspended in 1 mL of ethyl acetate:tetrahydrofuran:acetonitrile:methanol (50∶7.5∶15∶27.5). The sample was analyzed for the lycopene content using high pressure liquid chromatography (Agilent 1200) at 475 nm wavelength. The column used for lycopene analysis was Supelco C18 with 4.6×250 mm and 5 µm particle size. The flow rate was maintained at 1.5 mL/min. The mobile phase was a mixture of methanol:acetonitrile:ethyl acetate:n-hexane (7∶7∶2∶2). Lycopene quantification was carried out by using a standard calibration curve based on peak area (y = 44.62x –123.2, R^2^ =  0.998) by using 10 to 50 µg/mL standard lycopene (>98%; Shanghai Angoal Chemicals Co. China). The relative increase in lycopene content of each transgenic line was calculated by using the following formula:
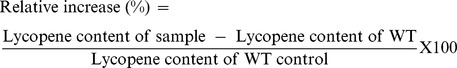



### Estimation of ascorbic acid and total phenolics from transgenic tomato fruits

Methanol extracts of three tomatoes from each *rolB* transgenic lines and control (*GUS* transformed and untransformed) were prepared for ascorbic acid and total phenolic analysis. Ascorbic acid (vitamin C) content in tomato fruit was determined using iodine titration method as described by [29]. Crude methanol extracts (10 mg) of transgenic tomatoes were dissolved in 1 ml of 100% methanol. The mixture was sonicated for 5 min and 150 µL of 1% starch indicator solution was added and mixed well. The samples were titrated against 0.1N iodine solution until an endpoint blue colour persists for more than 20 sec. Volume of the 0.1N iodine solution used in each titration was recorded and used for quantification of ascorbic acid in the sample (1 µL of 0.1N iodine used  = 8.8 µg of ascorbic acid). All the samples were analyzed in triplicate. The relative increase in ascorbic acid content of each transgenic line was calculated by using the following formula:
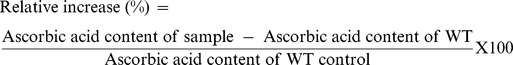



Total phenolic content of methanol extracts of transgenic and control tomatoes were determined with the help of Folin Ciocalteu assay as described by [30]. Crude methanol extracts of fruits were dissolved in 50% (v/v) aqueous methanol to a final concentration of 2 mg/mL. An aliquot of 200 µL of each sample was thoroughly mixed with 200 µL of the Folin Ciocalteu reagent. After 5 min 2 mL of 6% (w/v) Na_2_CO_3_ solution was added and total volume was made upto 5 mL with double distilled water. After incubation of 30 min at room temperature, absorbance was measured at 765 nm with the help of UV-vis spectrophotometer (Agilent technologies). Standard calibration curve was prepared by using various concentrations (0, 10, 20, 30, 40, 50 mg/L) of gallic acid. Total phenolics were expressed as mg gallic acid equivalent (GAE)/g fresh weight. All the samples were analyzed in triplicate. The relative increase in total phenolics of each transgenic line was calculated by using the following formula:
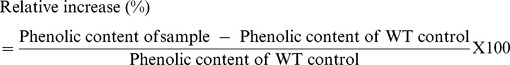



### Determination of antioxidant activity

The antioxidant activity of the fruit extracts from both transformed (*rolB* and *GUS*) and untransformed plants were determined by measuring their potential to scavenge the free radicals 2,2-diphenyl-1-picryl-hydrazyl (DPPH) according to the modified protocol of [31]. An amount of 3.2 mg of DPPH was dissolved in 100 mL of 82% aqueous methanol. Crude methanol extracts (15 mg/mL) were dissolved in 1.5 mL of 67% aqueous methanol and samples were sonicated in an ultrasonic bath for 5 min. DPPH solution (2.8 mL) was added to each vial along 200 µL aliquots of diluted methanol extract (10, 100, 1000 ppm). The mixture was vortexed and after one hour, absorbance was taken at 517 nm by using UV-vis spectrophotometer (Agilent Technologies). Aqueous methanol solution (82%) was used as blank while mixture of DPPH and methanol (14∶1 v/v) was taken as control. All the samples were analyzed in triplicate and their scavenging potential was calculated by applying following formula: 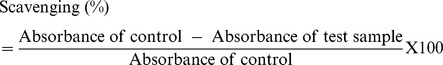



EC_50_ values of samples were calculated by means of graphical method. Moreover, the relative increase in antioxidant activity of each transgenic line was calculated by using the following formula:
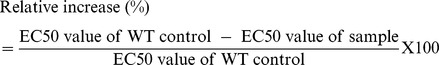



### Disease response assays of *rolB* transformed plants

The response of *rolB* gene transformants for pathogen infection was determined by using electrolyte leakage test and detached leaf assay.

Electrolyte leakage test was conducted on leaves from 24 plants of 9 homozygous *rolB* lines in the presence of 5 mM fusaric acid (Sigma) following the protocol described by [32]. One gram leaf discs (∼8 mm diameter) from different mature transgenic lines and control plants (*GUS* transformed and untransformed) were incubated in 25 mL of 3% sucrose solution supplemented with 5 mM fusaric acid and conductivity of solution was measured using digital conductivity meter. The data was statistically analyzed using MSTATC.

Resistance of *rolB* transformed plants was tested against two fungal strains (*F. oxysporum* and *A. solani*) by using detached leaf assay. Fungal strains were inoculated on 4% Sabouraud Dextrose Agar (SDA) medium and incubated at 28°C for two weeks till sporulation stage. Detached leaves were rinsed with sterile distilled water, blotted dry and cultured on SDA medium containing fungal cultures. Petri plates were sealed with parafilm and stored at 28°C for 7 days. Twenty four leaflets (8 leaflets from 3 plants) from each transgenic line were inoculated on each fungal strain. Experiment was performed with three replicates for each treatment. Visual observations were taken regularly for fungal growth and development of disease symptoms on leaves. After 7 days, the numbers of infected leaves were counted for each treatment and disease incidence was calculated as:
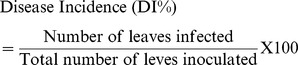



The total infected area of 24 leaves was measured by using optical planimeter and the percentage of their mean value was calculated and finally the disease severity was estimated according to the scale described by [33].

## Results

*Agrobacterium*-mediated tomato transformation was successfully used to produce transgenic tomato plants expressing *A. rhizogenes rolB* gene. Out of 266 cotyledons co-cultivated with *A. tumefaciens*, 102 regenerated shoots after six weeks on regeneration medium containing 100 mg/L kanamycin. Shifting of elongated shoots to rooting medium supplemented with 100 mg/L kanamycin resulted in root development in 61 shoots (Table S1). Similarly, 47 regenerated shoots from 100 explants that were transformed with p35SGUSint (control) were rooted on rooting medium containing 100 mg/L kanamycin.

### Confirmation of transgene integration

Out of 61 tested plants, 58 showed an expected PCR product of 779 bp for *rolB* gene (Figure 1B, data shown for six *rolB* lines). Transformation frequency was calculated on the basis of *rolB* PCR positive plants with respect to total number of co-cultivated explants and was found to be 21.80% (Table S1). In case of *GUS* transformed plants, PCR yielded amplification of 895 bp fragment of *GUS* gene while no such band was observed in WT plants (Figure 1B, data shown for six transformed lines). In case of *GUS* transformed plants, transformation efficiency was 37% (Table S1).

Southern blot analysis for *rolB* transformants showed that all the samples except negative control gave significantly high signals with DIG-labelled *rolB* probe, indicating the presence of *rolB* gene in these lines. It was observed that in one *rolB* transformed line RB I two copies of *rolB* gene were inserted in the tomato genome compared to rest, which contained only one copy (Figure 1C, data shown for six *rolB* lines). The band pattern was slightly different between different transgenic lines suggesting the independent transformation events. As expected, no band was observed in WT (negative control).

### Morphological analysis of *rolB* transgenic lines

Transgenic lines that exhibited stable integration of transgene and produced viable seeds were selected for further analyses. Seeds of the T_0_ transgenic tomato plants were germinated on selection medium to obtain transgenic T_1_ progeny. Results, as shown in Table S2, indicate that the transgene was inherited successfully in the T_1_ progeny of *rolB* gene transformed plants and followed the Mendelian ratio of 3∶1. One of the *rolB* expressing transgenic lines (RB I) exhibited the Mendelian segregation pattern of 15∶1. T_2_ generation was raised from the transgenic tomato seeds in the same way as described above. Homozygous lines from T_2_ progeny which showed 100% kanamycin resistance were selected for further analyses.

**Figure 2:**
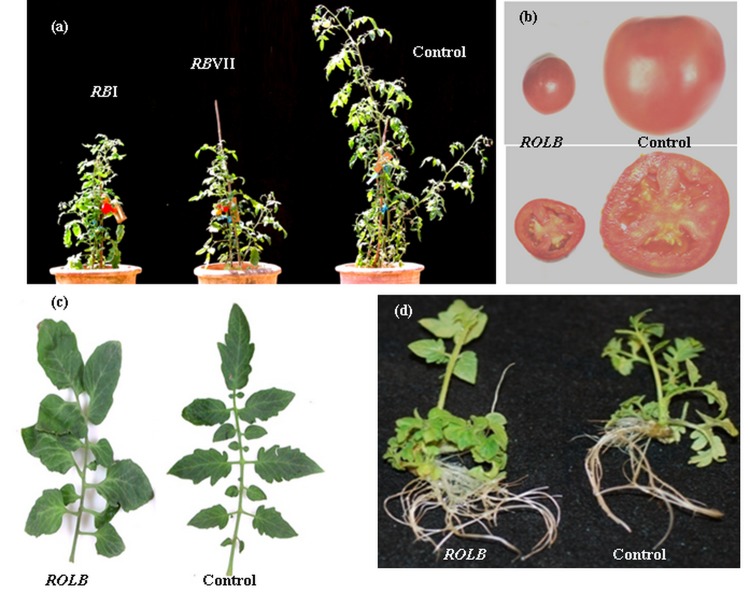
Morphological analysis of *rolB* transgenic tomato plants cv. Rio Grande. (a) mature plants (b) leaves (c) roots (d) tomato fruit (whole and TS).

The *rolB* expressing transgenic plants had a change in their vegetative growth and morphology when compared to WT tomato plants. However, no significant difference was observed between transformed control (*GUS* transformed plants) and WT tomato plants. All *rolB* transgenic lines were significantly shorter and produced smaller fruits when compared to WT plants (Figure 2a). The *rolB* expressing tomato plants had an average height of 42.3 cm as compared to 94 cm for WT plants. A reduction in apical dominance along with shorter internodal length was also observed in *rolB* expressing plants. Leaves were also smaller, less serrated and more oval in shape as compared to *GUS* control and WT. In some *rolB* transformed plants, mild wrinkled leaves were observed (Figure 2c). Interestingly, *rolB* expressing plants had profuse, long and hairy root system as compared to control (Figure 2d). The flowers were normal in size but some of them were infertile and did not produce fruits. Fruits that developed in *rolB* transgenic plants were smaller in size (Figure 2b) and matured earlier when compared to the fruits of control and WT plants. Number of fruits and their weight was calculated from 24 *rolB* expressing plants that were derived from nine independent transgenic lines. The *rolB* expressing tomato plants produced an average of 11 fruits per plant whereas control and WT plants produced and an average of 15 fruits per plant. Average fruit weight of *rolB* transgenic tomatoes was 38 g and was significantly lower than control/WT plants that had 74 g.

### Fruits of *rolB* transformed plants showed enhanced nutritional quality

**Figure 3:**
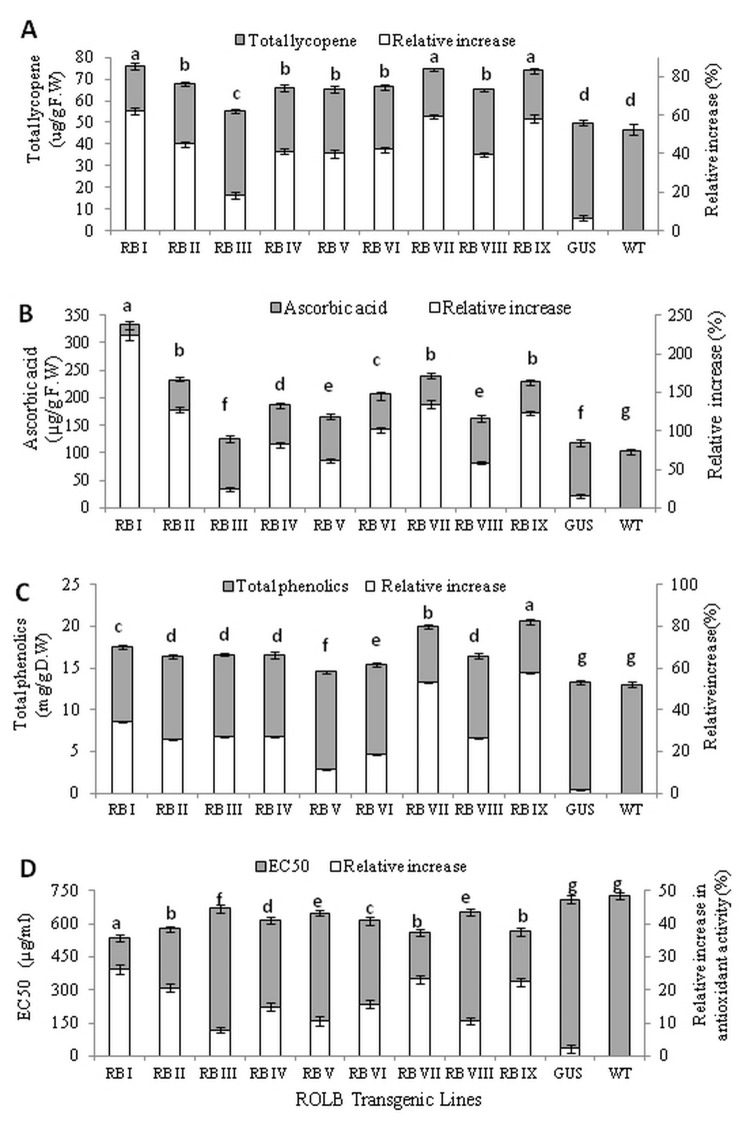
Biochemical analysis of *rolB* transformed tomato plants. (**A**) Determination of Lycopene content (**B**) Ascorbic acid content (**C**) Total phenolic content and (**D**) Antioxidant activity along with their relative increase in fruits of *rolB* transgenic lines of tomato. RB  =  *rolB* transformed; *GUS* =  *GUS* transformed control; WT  =  Wild-type (untransformed control). Data represents mean of three replicates. Any two values with same alphabet did not differ significantly at 5% probability level using LSD test.

To investigate the effect of *rolB* expression on important nutritional contents, biochemical analyses were performed on the fruits generated from *rolB* expressing plants. Strikingly, all the *rolB* expressing tomato lines showed significant increase (18 to 62%) in their lycopene content when compared to control and WT. The line RB I exhibited maximum increase of 62% in lycopene content (76 µg/g) while two other lines RB VII and RB IX showed an increase of 59% (74 µg/g) and 58% (74 µg/g), respectively in their lycopene content when compared to controls (Figure 3A). All the tested *rolB* expressing tomato fruits showed significantly higher (24 to 225%) ascorbic acid content than the controls. The highest relative increase (225%) in ascorbic acid level was observed for RB I with 333 µg/g ascorbic acid (Figure 3B). In addition, *rolB* expressing tomato fruits exhibited 11% to 58% increase in total phenolic content. Two *rolB* lines RB IX and RB VII exhibited maximum increase of 58% (21 mg/g) and 53% (20 mg/g), respectively, in their phenolic contents (Figure 3C). Considerable increase in the antioxidant activities was also observed for all *rolB* transformed plants as compared to controls. Highest relative increase (26%) in antioxidant activity was exhibited by RB I (EC_50_ = 536 µg/mL) followed by RB VII (EC_50_ = 558 µg/mL) and RB IX (EC_50_ = 564 µg/mL) which showed 23% and 22% increase in their antioxidant capacities, respectively (Figure 3D).

### Transgenic plants showed enhanced foliar tolerance to fungal pathogens

**Figure 4:**
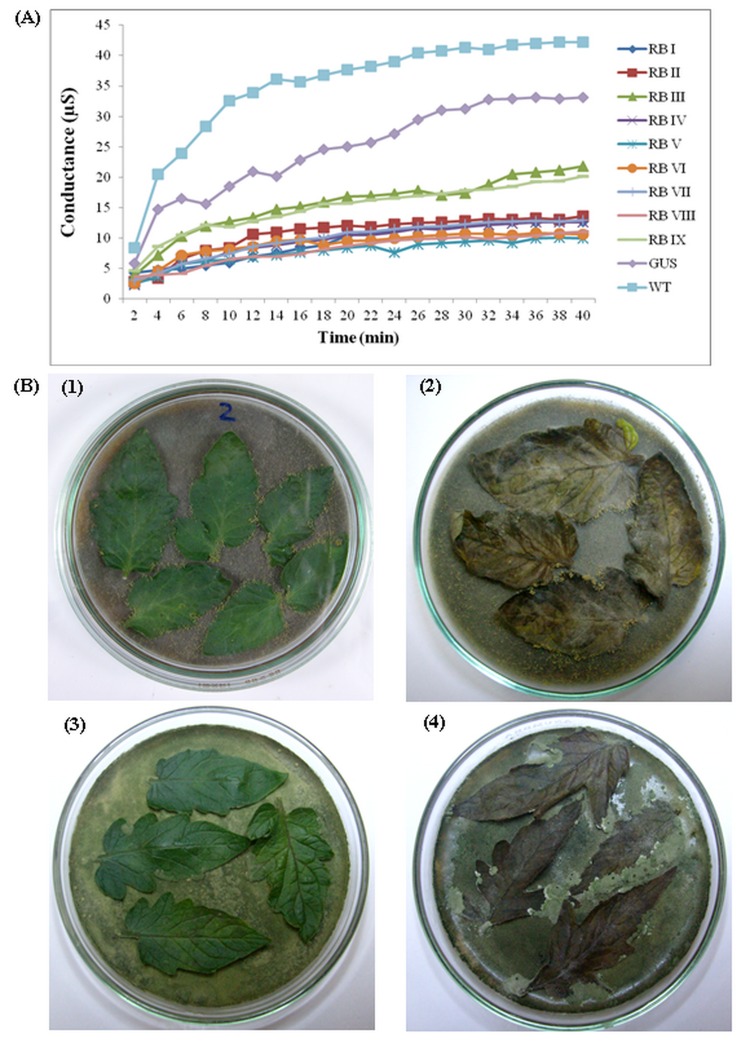
Disease response of *rolB* transformed tomato leaves. (**A**) Effect of *rolB* on disease response of leaves of transgenic tomato plants in ion leakage experiment (**B**) Detached leaf assay of *rolB* expressing tomato plants (1) Leaves of *rolB* transformed plants infected with *Alternaria solani* (2) Control leaves infected with *A. solani* (3) Leaves of *rolB* transformed infected with *Fusarium oxysporum* (4) Control leaves infected with *F. oxysporum*.

Analysis of data exhibited that the difference between *rolB* transgenic lines and controls (both untransformed wild type and GUS transformed) was highly statistically significant at 1% α level (P<0.01, Table 1). Results of electrolyte leakage assay showed that *rolB* expressing tomato plants exhibited moderate to high level of tolerance to the toxin, fusaric acid, when compared to control and WT tomato plants (Figure 4A). Less electrolyte leakage due to the toxin could be an indicative of enhanced defence response. Least electrolyte leakage was observed in *rolB* expressing tomato line RB V while the highest was observed in transgenic line RB III.

**Table 1:** Analysis of variance for electrolyte leakage.

Source	Degree of freedom	Sum of squares	Mean Square	F- Value	Probability
Replication	2	17.560	8.780	772636.3084	0.0000
Factor A (Time)	19	9726.731	511.933	45049844.6417	0.0000
Factor B (Samples)	10	43877.352	4387.735	386118309.4630	0.0000
AB	190	3255.279	17.133	1507698.7921	0.0000
Error	438	0.005	0.00014		
Total	659	56876.927			

Coefficient of variation (CV) =  0.2%.

Mature *rolB* expressing transgenic T_2_ plants were directly assayed for their defence response against two fungal pathogens (*A. solani* and *F. oxysporum*), by using detached leaves assay (Table 2). Interestingly, leaves of *rolB* expressing tomato plants exhibited moderate to high levels of tolerance to both fungal pathogens tested (Table 2; Figure 4B). It is evident from the results that all the tested *rolB* transgenic lines showed disease symptoms. However, they varied in disease incidence and severity level when compared to controls. In case of *A. solani*, DI ranged from 4.17% (RB IX) to 25% (RB III and RB IV) while DI varied from 4.17% (RB V) to 27.67% (RB III) for *Fusarium* wilt (Table 2). Moreover, the DS ranged from 1.75% (RB IX) to 16.75% (RB IV) for *A. solani* and 2.50% (RB V) to 18.75% (RB III) for *F. oxysporum*. Strikingly, Six *rolB* lines (RB I, RB II, RB V, RB VI, RB VII and RB VIII) displayed less than 10% disease severity for both *A. solani* and *F. oxysporum* and were considered resistant (R) against both these pathogenic fungi. Leaves of WT tomato plants showed very high DI (91.67 and 95.83%) and DS (67.88 and 59.75%) for *A. solani* and *F. oxysporum*, respectively. *GUS* transformed control tomato plants (*GUS*) were also susceptible to both these fungal pathogens since they had high percentages of DI (79.17 and 83.33%) and DS (63.75 and 52.50%) for *A. solani* and *F. oxysporum*, respectively.

**Table 2:** Disease response of leaves from *rolB* gene transformed plants against two fungal pathogens on the basis of detached leaf assay.

Tomato lines		*Alternaria solani*			*Fusarium oxysporum*		
		DI* (%)	DS$ (%)	Host Responseα	DI (%)	DS (%)	Host Responseα
***rolB***	RB I	16.68	6.25	R	12.50	7.25	R
	RB II	20.85	7.25	R	20.83	8.25	R
	RB III	25.00	13.75	MR	27.67	18.75	MR
	RB IV	25.00	16.75	MR	12.50	6.25	R
	RB V	20.85	6.50	R	4.17	2.50	R
	RB VI	20.85	9.50	R	8.33	4.00	R
	RB VII	12.50	3.75	R	12.50	7.25	R
	RB VIII	8.34	2.75	R	8.33	6.00	R
	RB IX	4.17	1.75	R	16.67	13.75	MR
**Control**	*GUS*	79.17	63.75	S	83.33	52.50	S
	WT	91.67	67.88	S	95.83	59.75	S

Each value is an average of three replicates of 24 leaflets each (8 leaflets ×3 plants).

^*^ DI% represents the disease incidence percentage.

$ DS% represents the disease severity percentage.

α Host response representing the level of resistance or susceptibility against a pathogen.

R  =  Resistant (1–10% DS), MR  =  Moderately resistant (11–25% DS), S  =  Susceptible (51–75% DS).

## Discussion

Improvement of fruit quality is desirable for important cultivated crops that are consumed in human diet. Tomato fruit is a rich source of many compounds such as antioxidants, which are important for human health. Antioxidants have a potential *rol*e in preventing and repairing damages caused by oxidative stress [34]. Since individual *rol* genes of *A. rhizogenes* are considered as potential enhancers of secondary metabolism in transformed plant cells and are capable of increasing the biosynthesis of secondary metabolites [4], we were interested to investigate the effect of *rolB* gene on the production of secondary metabolites. We measured lycopene, ascorbic acid and phenloic contents of different lines of *rolB* expressing tomato plants as well as antioxidant activities of these transgenic lines. We found that tomato plants transformed with *rolB* gene exhibited enhanced production of secondary metabolites in fruits. Various *rolB* transgenic tomato fruits differed significantly for their lycopene, ascorbic acid and phenolic contents as well as antioxidant activity compared to control plants. Among different transgenic lines, RB I showed the highest increase in lycopene and ascorbic acid contents as well as exhibited highest antioxidant activity. Compared to other *rolB* expressing lines, this increased level in RB I could be attributed to the insertion of two copies of *rolB* gene in the genome, as evident from Southern blot hybridization. In case of phenolic content, highest increase was observed in transgenic line RB IX. A 100 fold increase in resveratrol production has been reported in *Vitis amurensis rolB* transformed plants [35]. Similarly, *Rubia cordifolia* calli transformed with *rolB* gene also showed enhanced antioxidant activity and suppression of reactive oxygen species [5], [36]. Thus, it can be suggested that expression of *rolB* gene in transformed tomato plants boosted the antioxidant activities of fruits by synergistically altering the secondary metabolism that resulted in enhanced production of lycopene, ascorbic acid and total phenolic contents.

Disease responses of the leaves of *rolB* transformed plants were evaluated by using electrolyte leakage test and detached leaf assay. Electrolyte leakage is an important indicator linked with the hypersensitive response in plants reacting to pathogen assault [37]. We estimated ion leakage from leaf discs after treating them with a solution of fusaric acid (a toxin produced by *Fusarium oxysporum*). The results showed that leaves from *rolB* gene transformed tomato plants exhibited higher degree of tolerance to the toxin as compared to both *GUS* transformed and WT tomato plants. The results also revealed a significant positive correlation between the quantity of electrolyte loss and the extent of plant susceptibility to pathogens. Some earlier reports have shown that tomato plants transformed with *rolD* gene exhibited enhanced tolerance against fusaric acid [32]. In general, loss of electrolyte is associated with disruption of cell membrane due to toxins or enzymes resulting in remarkable increase in shifting of K^+^ and H^+^ ions across the cell membrane [38].

In detached leaf assay, typical disease symptoms like wilting, chlorosis and necrosis were observed on leaves of all transgenic lines of tomato. However, the degree of diseases incidence and severity in *rolB* gene transformed plants was significantly lower than WT and *GUS* transformed control plants. In case of *A. solani*, seven out of nine *rolB* transformed lines of tomato exhibited complete resistance while two were moderately resistant. Among different *rolB* expressing lines, leaves of RB IX showed highest tolerance to *A. solani* infection. Similarly, in case of *F. oxysporum*, leaves of seven *rolB* transformed lines showed complete tolerance while two lines exhibited moderate tolerance. In contrast to *A. solani*, *rolB* transgenic line RB V exhibited highest foliar tolerance to *F. oxysporum*. One possible reason for such improved response could be the defence related compounds such as phenolics [39]. The variable foliar tolerance of different transgenic lines towards *A. solani* and *F. oxysporum* might reflect the species specific response of plants against pathogens. The effect of *rolD* and *rolC* genes on plant disease resistance has been reported in tomato [32] and strawberry [40], respectively. It is evident that different *rol* genes have different effects on the growth and metabolism of plants [41]. It has been shown that plant defence response in *rol* genes transformed plants is activated by a protein kinase/phosphatase cascade through signal transduction [42], [43] and phytoalexin synthesis by Ser/Thr phosphatases [44], [45], [46]. The expression of *rol* genes under CaMV35S promoter also modifies calcium balance and stimulate the PR-2 protein synthesis [4] leading to phytoalexins synthesis involved in plant defence [47]. Different reports have shown that expression of *rol* genes also results in altered expression and activity of calcium-dependent protein kinases (*CDPK*) genes in transformed cells, leading to increase in the level of resistance of these transgenic plants against pathogens [48], [49].

Although, among the *rol* genes, *rolB* is the most powerful activator of secondary metabolism, it also has growth suppressing effect [4]. In this report, we studied the effect of *rolB* on the morphology of transformed plants. The *rolB* expressing tomato plants showed reduction in plant height, smaller and wider leaves with mild wrinkling. Reduced apical dominance and short inter-nodal distances were also the factors influencing the plant height. Although, average number of flowers were approximately same in *rolB* transformed plants and controls (*GUS* transformed and WT), flower infertility resulted in lesser yield. It has been reported that loss of pollen viability in *rolB* transformed plants inhibits fruit development [50]. The fruits produced on *rolB* expressing plants were also smaller in size and ripening time was shorter as compared to control plants. Among different *rolB* expressing lines, RB I was the most affected line, with least average plant height and lowest average number of fruits. It has been reported that expression of *rolB* gene increases the secondary metabolites [5]; however, excessive expression of gene has a growth suppressive effect. In general, in most cases, nuclear transformation is accompanied with detrimental effects on plants, which can be overcome by chloroplast-based expression of foreign genes [51]. In addition, the use of pathogen-inducible promoters can also be considered. In case of *rolB* transformed plants, the enhanced expression of *rolB* gene can be a reason for this phenomenon as two copies of *rolB* gene were inserted in RB I, which in turn might have affected the morphology more severely compared to other transgenic lines. Due to insertion of *rolB* gene, variations in various plant morphological characters have been reported in a number of plant species such as tobacco and tomato [14], [15], [23], *Antirrhinum* [20], Rose [17], Pear [52], grape [53] and soybean [21]. Overall, these changes could be attributed to increase in sensitivity of *rolB* transformed cells toward auxin [8], [9], increased tyrosine phosphatase activity that alters the signal transduction pathway of the hormone [54], H^+^ ATPase and ionic imbalance [8]. All these changes synchronize the metabolic pathways leading to variation in flower and fruit development [21]. Variations in flower/fruit number and phenotype are also elemental demarcations based on active auxin release that collectively increases the production of endogenous gibberellins [55].

In conclusion, the data obtained in the present study suggest that the activation of secondary metabolism is modulated by the signals provided by *rolB* gene expression in the transformed tomato plants. Transgenic plants show altered morphology, which could be directly linked with the inserted copy number of *rolB* gene and consequently to its enhanced expression. We report the possible role of *rolB* gene in increasing the level of different antioxidants such as ascorbic acid and lycopene that can benefit human health. Moreover, defence response of *rolB* expressing tomato plant leaves was significantly enhanced against two fungal pathogens *A. solani* and *F. oxysporum*, as evident from pathogenicity assays. Taken together, these data can aid in the development of nutritionally improved tomato plants with improved foliar resistance against fungal infections.

## Supporting Information

Table S1 **Transformation summary of tomato cv. Rio Grande.** ^α^ Percentage of PCR positive plants divided by total number of co-cultivated explants.
(DOC)

Table S2 **Inheritance and segregation of transgene in T_1_ progeny of different** ***rolB*** **transgenic lines.** Tabulated **χ** ^2^ value for 1 degree of freedom at 5% probability is 3.84. All **χ** ^2^ values indicate a good fit to the expected Mendelian segregation ratio as the calculated **χ** ^2^-value is less than the **χ** ^2^ table value.
(DOC)
