# Supplementary material for: Agrobacterium-Mediated Transformation of Tomato with rolB Gene Results in Enhancement of Fruit Quality and Foliar Resistance against Fungal Pathogens
Source: PLoS One. 2014 May 9;9(5):e96979. doi: 10.1371/journal.pone.0096979 (PMC4016209; doi:10.1371/journal.pone.0096979)
Supplement: Table S1 — Transformation summary of tomato cv. Rio Grande. α Percentage of PCR positive plants divided by total number of co-cultivated explants. (DOC) [file pone.0096979.s001.doc]

**Table S**1: Transformation summary of tomato cv. Rio Grande

| ***Agrobacterium* strain (Construct)** | **No. of co-cultivated explants** | **No. of regenerated shoots on selection medium** | **No. of rooted shoots on selection medium** | **PCR positive plants with respective gene** | **Transformation efficiency (%)^α^** |
| --- | --- | --- | --- | --- | --- |
| LBA4404 with pLBR30 (*rolB*) | 266 | 102 | 61 | 58 | 21.80 |
| LBA4404 with p35SGUSint (*GUS*) | 100 | 63 | 47 | 37 | 37 |
