# Supplementary material for: Agrobacterium-Mediated Transformation of Tomato with rolB Gene Results in Enhancement of Fruit Quality and Foliar Resistance against Fungal Pathogens
Source: PLoS One. 2014 May 9;9(5):e96979. doi: 10.1371/journal.pone.0096979 (PMC4016209; doi:10.1371/journal.pone.0096979)
Supplement: Table S2 — Inheritance and segregation of transgene in T1 progeny of different rolB transgenic lines. Tabulated χ 2 value for 1 degree of freedom at 5% probability is 3.84. All χ 2 values indicate a good fit to the expected Mendelian segregation ratio as the calculated χ 2-value is less than the χ 2 table value. (DOC) [file pone.0096979.s002.doc]

**Table S2: Inheritance and segregation of transgene in T_1_ progeny of different *rolB*** transgenic lines

| **Transgenic Lines** | **Total T_1_ seedlings** | ***NPTII* Resistant seedlings** | ***NPTII* Susceptible seedlings** | **Expected segregation ratio** | **χ^2^** | **P value** |
| --- | --- | --- | --- | --- | --- | --- |
| RB I | 13 | 12 | 1 | 15 : 1 | 0.046 | 0.829 |
| RB II | 20 | 14 | 6 | 3 : 1 | 0.266 | 0.605 |
| RB III | 25 | 17 | 8 | 3 : 1 | 0.653 | 0.418 |
| RB IV | 17 | 14 | 3 | 3 : 1 | 0.490 | 0.483 |
| RB V | 25 | 16 | 9 | 3 : 1 | 2.44 | 0.117 |
| RB VI | 33 | 23 | 10 | 3 : 1 | 0.494 | 0.481 |
| RB VII | 19 | 14 | 5 | 3 : 1 | 0.017 | 0.894 |
| RB VIII | 27 | 19 | 8 | 3 : 1 | 0.308 | 0.578 |
| RB IX | 20 | 15 | 5 | 3 : 1 | 0 | 1 |
